# Enhancing the Potential of Microhaplotypes for Forensic Applications: Insights from Afghan and Somali Populations

**DOI:** 10.3390/genes16050532

**Published:** 2025-04-29

**Authors:** Pedro Rodrigues, Nádia Pinto, Tess Otterlund, Carina G. Jønck, Maria João Prata, Claus Børsting, Vania Pereira

**Affiliations:** 1 Section of Forensic Genetics, Department of Forensic Medicine, Faculty of Health and Medical Sciences, University of Copenhagen, Frederik V’s Vej 11, DK-2100 Copenhagen, Denmark; pedro.rodrigues@sund.ku.dk (P.R.); tfso.otterlund@gmail.com (T.O.); carina.joenck@sund.ku.dk (C.G.J.); vania.pereira@sund.ku.dk (V.P.); 2 Instituto de Investigação e Inovação em Saúde (i3S), 4200-135 Porto, Portugal; npinto@i3s.up.pt (N.P.); mprata@ipatimup.pt (M.J.P.); 3 Institute of Molecular Pathology and Immunology, University of Porto (IPATIMUP), 4200-135 Porto, Portugal; 4 Faculty of Sciences, University of Porto (FCUP), 4200-135 Porto, Portugal; 5 Centre of Mathematics, University of Porto, 4200-135 Porto, Portugal

**Keywords:** microhaplotypes, massively parallel sequencing, forensic and population parameters, ancestry, kinship testing

## Abstract

Microhaplotypes (MHs) are a novel class of genetic markers, exhibiting features that position them as an alternative to STRs and SNPs in addressing challenges commonly encountered in forensic investigations. Additionally, MHs can also offer valuable insights for ancestry inference. However, due to the novelty of MHs, extensive research in different global populations is required before implementation in forensic casework and general research. In this study, individuals from Afghanistan and Somalia were characterized with the Ion AmpliSeq™ MH-74 Plex Research Panel previously developed for forensic genetic purposes. A total of 84 Afghan and 89 Somalian samples were sequenced on the Ion GeneStudio™ S5 System. This led to the identification of 32 and 42 single nucleotide variants in the Afghan and Somalian populations, respectively, that were not included in the former MH definitions. Most of the observed variants were considered to be rare occurrences, being observed one or two times in the dataset. The average values of the effective number of alleles (A_e_) were 3.7 for Somalia and 3.6 for Afghanistan—pointing to elevated intrapopulation diversities compared to Europeans. Other parameters (H_o_, H_e_, PIC, PD, and PE) consistently showed higher average values in the Afghans and Somalis compared to the previously studied populations. PCA and STRUCTURE analyses with 1000 Genomes samples assigned the Somalis to a different cluster than the other sub-Saharan African populations. The analyses also showed higher European and East Asian co-ancestry in the Afghans than in the remaining South Asian populations. The capability of the MH-74 plex to address common kinship problems was evaluated through computational simulations, considering generic thresholds differing by one order of magnitude to assess the FDRs. The median LR > 10^13^ for true siblings when the hypotheses ‘full siblings’ and ‘unrelated individuals’ were compared. As expected, the median LRs were much lower for simulated half-siblings and cousins. This work evaluated the forensic potential of MHs in understudied populations. Overall, the studied panel was versatile and capable of being applied in different forensic applications.

## 1. Introduction

In the field of forensic genetics, Short Tandem Repeats (STRs) are usually considered the first choice of loci in the routine setting [1,2,3]. Among the features that give them preference over other genetic loci are their multi-allelic nature, their high discrimination power, and the ease of amplification and genotyping using capillary electrophoresis (CE)-based methods [4,5,6]. However, STRs have a number of limitations that can affect the interpretation of the profiles. Some of the disadvantages stem from (1) the large sizes of the amplified fragments, which restrict the number of loci that can be multiplexed in a single CE assay, (2) the length variation, which leads to preferential amplification of shorter alleles when working with degraded samples, and (3) the repetitive nature, which causes polymerase slippage during the Polymerase Chain Reaction (PCR) and creates stutter artifacts that may complicate the interpretation of the results [5,6,7,8].

Advancements in massively parallel sequencing (MPS) technologies enabled the exploration of new genetic markers with potential applications in forensic casework. This led to the emergence of microhaplotypes (MHs), which are relatively short-sized (<300 bp) genetic loci consisting of two or more linked single nucleotide polymorphisms (SNPs) transmitted as a block [9]. MHs share advantages with STRs, such as their multi-allelic nature and high discrimination power, but unlike STRs, MHs present neither repetitive regions prone to stutters nor significant length variations between different alleles from the same locus. Additionally, short MH loci may be selected to allow amplification of the loci on short PCR fragments [10]. Therefore, MHs are more suitable than STRs for forensic analyses of degraded DNA [11,12] and unbalanced mixtures [5,6,13]. In combination, the attributes of MHs make them appropriate for many different forensic investigations, including human identification, kinship testing, and biogeographic ancestry inference [14].

The purpose of this study was to investigate the potential applications of MHs in population samples from Somalia and Afghanistan—two populations for which data remain scarce. The analysis was carried out using the MH-74 plex panel described in Oldoni et al. [15] to perform comparative analyses with previously reported data for the same panel in samples from other populations [16,17,18,19].

## 2. Materials and Methods

### 2.1. Population Samples and Extraction

Anonymous biological material from 89 Somalis and 84 Afghans, all unrelated, were selected from ‘Section of Forensic Genetics anonymous collection of samples’ (reference number 004-0065/21-7000). Buccal swabs collected on Whatman^®^ FTA^®^ cards (Merck KGaA, Darmstadt, Germany) and blood samples were extracted using the EZ1^®^ Advanced XL robot (Qiagen, Hilden, Germany) along with the EZ1^®^ DNA investigator Kit (Qiagen, Hilden, Germany). DNA quantification was performed using the Qubit^®^ dsDNA HS Assay Kit (Thermo Fisher Scientific, Waltham, MA, USA) and the Qubit^®^ 3.0 Fluorometer (Life Technologies, Carlsbad, CA, USA), or the Quantifiler™ Trio DNA Quantification Kit with the Applied Biosystems™ 7500 Real-Time PCR System (Thermo Fisher Scientific, Waltham, MA, USA).

The study follows the policies from the Danish National Center for Ethics (https://nationaltcenterforetik.dk/; accessed on 11 February 2025) and the Danish Research Ethics Committees (https://videnskabsetik.dk/; accessed on 1 February 2025) and complies with the rules of the General Data Protection Regulation (Regulation (EU) 2016/679).

### 2.2. Amplification

Seventy-four loci from the custom Ion AmpliSeq™ MH-74 Plex Research Panel described by Oldoni et al. [20] and available in https://ampliseq.com (accessed on 1 April 2025) were amplified in 10 μL reaction volume 5 µL of primer mix (Thermo Fisher Scientific, Waltham, MA, USA), 2 µL of 5X Ion Ampliseq™ HiFi Mix (Thermo Fisher Scientific, Waltham, MA, USA), 1 ng/µL of gDNA, and the volume was completed with molecular grade water]. The samples were run in an Applied Biosystems^®^ Veriti^®^ 96-Well Thermal Cycler (Thermo Fisher Scientific, Waltham, MA, USA) with the following cycling conditions: 99 °C, 2 min; (99 °C, 15 s; 60 °C, 4 min) 24 cycles; 10 °C on hold.

### 2.3. Library Building and Sequencing

Libraries were prepared using FuPa reagent and the Ion Express™ Barcode X kit (Thermo Fisher Scientific, Waltham, MA, USA), applying half of the recommended volume. Library purification was carried out using magnetic beads [Agencourt^®^ AMPure^®^ XP Reagents (Agencourt, Beverly, CA, USA)], and quantified according to the Qubit dsDNA High Sensitivity Assay Kit User Guide (Thermo Fisher Scientific, Waltham, MA, USA) using the Qubit^®^ 4.0 Fluorometer (Life Technologies, Carlsbad, CA, USA). The purified libraries were diluted with nuclease-free water to a final concentration of 80 pM or 100 pM.

Ion Chef™ instrument (Thermo Fisher Scientific, Waltham, MA, USA) and Precision ID Chef Reagents (Thermo Fisher Scientific, Waltham, MA, USA) were used to perform clonal amplification by emulsion PCR and chip loading. Between 30 to 35 libraries were included per Ion 530™ sequencing chip and sequenced on the Ion GeneStudio S5™ system (Thermo Fisher Scientific, Waltham, MA, USA) using the Precision ID S5™ Sequencing Kit (Thermo Fisher Scientific, Waltham, MA, USA).

The FASTQ files were analysed with an in house-developed Python script called MHinNGS [21] (https://hub.docker.com/r/bioinformatician/mhinngs (accessed on 1 April 2025)) for quality control and genotype calling. The minimum coverage threshold for genotype acceptance was set to 100 reads, and the heterozygote balance (Hb) threshold was set to 0.33 < Hb < 3.3. MHinNGS utilizes a configuration file containing all of the detailed information for each locus and the criteria for calling the MH genotypes. Few changes were made to the default criteria in the configuration file [18]. The ‘noise filter’ parameter was increased from 1% to a maximum of 2.5% for seven loci, while the ‘slide’ function was changed from two to a maximum of five in 34 loci (Supplementary Table S1). Two analysts examined the MHinNGS output files independently, ensuring the accurate determination of the genotype calls.

### 2.4. Population Data Analysis and Forensic Statistical Parameters

Arlequin ver 3.5.2 [22] was used to calculate the allele counts and frequencies, observed (H_o_) and expected (H_e_) heterozygosity, as well as statistics regarding linkage disequilibrium (LD), Hardy–Weinberg equilibrium (HWE), and pairwise *F*_ST_ distances between Danes, Greenlanders [18], Somalis, and Afghans after 10,000 permutations. The statistical significance was set to α = 0.05. Whenever appropriate, the statistical significance was adjusted through the Bonferroni correction for multiple tests. The polymorphism information content (PIC), power of discrimination (PD), and power of exclusion (PE) were computed using the online tool STRAF 2.1.5 [23] (https://straf-p7bdrhm3xq-ew.a.run.app/ (accessed on 1 April 2025)). The combined power of discrimination (CPD) and the combined power of exclusion (CPE) of a set of k loci were determined based on the respective formulas, representing as ${PD}_{i}$ and ${PE}_{i}$ the power of discrimination and the power of exclusion, respectively, of the locus *i*: $CPD=1–\prod_{i=1}^{k}1–{PD}_{i}$; and $CPE=1–\prod_{i=1}^{k}1–{PE}_{i}$. The effective number of alleles (A_e_) was determined using the formula described by Crow and Kimura [24]:

$$Ae=\frac{1}{1-H_{e}}$$

Principal Component Analysis (PCA) was performed using the package *hierfstat* ver 0.5.11 [25] in R following the model presented by Patterson et al. [26]. STRUCTURE v.2.3.4 [27] was used for analyses comprising ten iterations per K value (K ranging from 4 to 8), with a burn-in of 100,000 steps and 10,000 MCMC steps utilizing correlated allele frequencies following the admixture model. In addition to the studied populations and the Danish and Greenlandic groups from Tomas et al. [18], a total of 2504 samples distributed across 26 populations from the 1000 Genomes Phase 3 database [28] were used for the PCA and STRUCTURE analyses (African Ancestry in SW USA—ASW; African Caribbean in Barbados—ACB; Bengali in Bangladesh—BEB; British From England and Scotland—GBR; Utah residents with Northern and Western European ancestry from the CEPH collection—CEU; Chinese Dai in Xishuangbanna, China—CDX; Colombian in Medellín, Colombia—CLM; Esan in Nigeria—ESN; Finnish in Finland—FIN; Gambian in Western Division—Mandinka—GWD; Gujarati Indians in Houston, Texas, USA—GIH; Han Chinese in Beijing, China—CHB; Han Chinese South—CHS; Iberian Populations in Spain—IBS; Indian Telugu in the U.K.—ITU; Japanese in Tokyo, Japan—JPT; Kinh in Ho Chi Minh City, Vietnam—KHV; Luhya in Webuye, Kenya—LWK; Mende in Sierra Leone—MSL; Mexican Ancestry in Los Angeles CA USA—MXL; Peruvian in Lima Peru—PEL; Puerto Rican in Puerto Rico—PUR; Punjabi in Lahore, Pakistan—PJL; Sri Lankan Tamil in the UK—STU; Toscani in Italia—TSI; Yoruba in Ibadan, Nigeria—YRI). 

New alleles identified in this study were subcategorized as ‘rare’ if they occurred one or two times across the entire sample set. Due to their low occurrence in the dataset, these rare variants were not included in the MHinNGS configuration file as allele-defining SNPs of the MH. However, they were still considered for all of the analyses presented.

### 2.5. Kinship Analyses

Familias software ver 3.3.1 [29] was used to simulate the pairwise genotypic configurations of individuals considering the allele frequency data for the Somali and Afghan individuals, assuming no mutations, no population substructure, no silent alleles, nor allele dropouts. Acknowledging that non-zero parameters would influence the statistical outcomes, the assumption of their absence was justified with a thorough, multi-faceted analysis considering both practical and scientific aspects. The practical aspects are related to the fact that similar studies also make these assumptions; thus, the comparison of the results for the performance of the different kits is only possible under the same methodology. In addition, the lack of accurate estimates for these parameters justifies the caution toward including them in the calculations. Finally, even if proper mutation estimates were available, the extraordinary computational demand needed to include the biologically realistic mutation model ‘Extended Stepwise’ in simulations prevents its inclusion. Although it should be considered in casuistic analyses for specific cases, the statistical impact of mutations when analyzing a batch of samples showed little general impact [30]. On the other hand, population substructure is expected to be low in modern populations, and the methodological-related parameters are also expected to have little impact, especially when analyzing reference samples. In any case, we concur that these parameters should be properly estimated and included in the casuistic setting where specific genotypic configurations, hypotheses, and sample conditions are analyzed. Three kinship scenarios were considered, assuming as the main hypothesis (H1) that the pair of individuals being related either as i. full siblings, ii. half-siblings, or iii. first cousins, while the alternative hypothesis (H2) considered that the individuals were unrelated. For each kinship scenario, 2 × 30,000 pairwise genotypic configurations were simulated, considering both the main and the alternative hypotheses (seed = 12345). The weight of the evidence was calculated as likelihood ratios (LRs) comparing the probability of obtaining the evidence, E, considering either the main hypothesis (H1) or the alternative hypothesis (H2): LR=P(E|H1)/P(E|H2).

For each kinship scenario, two sets of data were considered: one where the pairwise genotypic configurations were simulated assuming that the individuals were related such as described in the main hypothesis, and the other where the individuals had been simulated as unrelated. Graphical representation of the LR results was obtained through an in-house R script. The percentage of simulations where related and unrelated individuals were correctly identified based on the evidence (LR>T for related individuals and LR<1/T for unrelated individuals) and incorrectly identified based on the evidence (LR<1/T for related individuals and LR>T for unrelated individuals), as well as the percentage of inconclusive results (1/T<LR<T), were calculated, assuming different LR thresholds, *T* = {1, 10, 10^2^, 10^3^, 10^4^}. These are generic thresholds *T*, differing by one order of magnitude, used to provide a visualization of the LR distributions obtained considering the simulated profiles given a specific population (or in other words, allele distributions) and a kinship problem comparing two alternative hypotheses.

## 3. Results and Discussion

### 3.1. Sequencing Results

Overall, the sequencing yielded a low rate of missing data, with only 34 out of 6586 possible genotypes (0.52%) not called for the Somalis, and 8 out of 6216 possible genotypes (0.13%) not called for the Afghans. Compared with previous results reported for Danes and Greenlanders [18], typed with the same panel in the same laboratory, the Afghan and Somali populations exhibited a higher percentage of genotypes that were not called, which might be due to the generally lower quality of these samples. 

### 3.2. Reported Variants and Rare Alleles

From the output files generated by MHinNGS, it was possible to identify additional variants on the amplified fragments in addition to the ones that were defined as part of the MHs. 

A total of 67 variants, not present in the original allele-defining SNPs of the loci, were identified in the Somali and Afghan samples (Supplementary Table S2). Of those novel variants, most were observed in the Somalis, with 42 variants (21 rare variants), whereas 32 variants (23 rare variants) were reported in the Afghan samples. The reported variants led to the identification of 89 new alleles. Of all of these variants, 27 were reported in more than 2 chromosomes, and consequently, they were incorporated as allele-defining SNPs of the respective MHs.

### 3.3. Population and Forensic Parameters

Microhaplotype allele frequencies for both populations are shown in Supplementary Table S3, whereas allele counts, A_e_, H_o_, H_e_, PIC, PD, and PE are provided in Supplementary Table S4. To facilitate comparisons between the two populations characterized in this study as well as those from Denmark and Greenland, two MHs (mh03KK-150 and mh09KK-033) were removed from the analyses due to locus dropouts and inconsistent results identified in the study conducted by Tomas et al. [18]. Of note, the variants regarded as ‘rare’ in Tomas et al. were considered for the forensic statistical parameters. 

The forensic statistical parameters can be found in Supplementary Table S4. One monomorphic locus (mh16KK-053; haplotype CT) was reported for the Somalis. The same allele (CT) was found to be fixed in all the sub-Saharan African populations from 1000 Genomes. No statistically significant deviations from Hardy-Weinberg equilibrium were observed in the Afghans and Somalis after Bonferroni correction for multiple tests (Supplementary Table S5). In both populations, the same pair of MHs (mh05KK-122 and mh05KK-123) showed to be in significant LD after Bonferroni correction. For the CPE and CPD computations, and for the kinship simulations (see below), mh05KK-123 was excluded based on the criterion of retaining the locus with the higher A_e_ in the pair. 

The allele count per locus highlighted the intrapopulation variation in the studied populations. The Somali population had the highest allele count per locus, presenting an average of 6.6 alleles per locus, whereas the Afghans, Danes, and Greenlanders exhibited an average of 6.1, 5.8, and 5.4, respectively. The locus mh21KK-315 was the most polymorphic in the studied populations, presenting 21 alleles in the Somalis and 17 alleles in the Afghans. This locus was also the most polymorphic one in the Danes and Greenlanders, reporting 15 and 13 alleles, respectively. The H_e_ in the Somalis, excluding the monomorphic locus, ranged from 0.118 (mh06KK-031) to 0.890 (mh21KK-315), with a mean of 0.661. In the Afghans, the H_e_ ranged between 0.124 (mh15KK-095) and 0.872 (mh02KK-134), presenting a mean value of 0.682, which was higher than the mean H_e_ value observed in the Somalis. The highest and lowest PIC values were observed in the same loci as the highest and lowest H_e_ values, spanning from 0.113 to 0.875 in the Somalis and 0.118 to 0.853 in the Afghans, with mean values of 0.614 and 0.630, respectively. The PD values ranged from 0.223 (mh06KK-031) to 0.968 (mh06KK-008) in the Somalis and 0.216 (mh15KK-095) to 0.960 (mh13KK-218) in the Afghans. The PE values in the Somalis ranged from 0.012 (mh06KK-031) to 0.788 (mh11KK-180 and mh21KK-324), and in the Afghans, they ranged from 0.010 (mh15KK-095) to 0.709 (mh13KK-218 and mh19KK-299). For the Somalis, the CPD and CPE values for this kit were 1–1.05 × 10^−59^ and 1–2.08 × 10^−19^, respectively, and for the Afghans, they were 1–1.76 × 10^−60^ and 1–7.16 × 10^−18^. Both parameters were higher in the studied populations than in the Danes and Greenlanders (Supplementary Table S6). Based on the CPD and CPE values, this assay exhibited greater effectiveness in differentiating individuals and excluding a falsely alleged parent than other commonly used forensic assays, e.g., the Precision ID GlobalFiler™ NGS STR Panel v2 (CPD = 1–3.18 × 10^−35^ and CPE = 1–1.85 × 10^−15^) in Japanese individuals [31] and the ForenSeq™ DNA signature Prep Kit (CPD = 1–3.3 × 10^−53^ and CPE = 1–1.14 × 10^−15^; only considering autosomal loci) in admixed Mexican individuals [32]. The same parameters were compared to different populations, sequenced with the same assay, from China (Chengdu Han, Chengdu Tibetan, and Liangshan Yi) [17] and the USA (African-descent, European-descent, Hispanic, and East Asian-descent) [16]. For all of the parameters, the Chinese populations obtained lower values (Supplementary Table S6). Regarding the U.S. populations, the Hispanic and African-descent groups reported similar H_e_ values (0.665 and 0.656, respectively) compared to the Somalis and Afghans; meanwhile the European- and East Asian-descent groups presented, correspondingly, 0.610 and 0.595. Additionally, the CPD and CPE results were closer to the ones obtained for the Hispanic- and African-descent groups than to the European-descent and East Asian-descent groups (Supplementary Table S6). The average number of alleles per locus was lower in the populations from the U.S. The exclusive use of the originally defined SNPs described for these MH loci may be the underlying cause. 

A_e_ was also assessed for all loci and averaged for each population (Supplementary Table S4). A_e_ is a statistical parameter that provides an estimate of the expected number of equally frequent alleles given the heterozygosity of a locus. A_e_ is commonly considered as the most informative parameter for the selection of microhaplotypes for forensic purposes [33]. The Somalis and Afghans presented higher average A_e_ values, 3.7 and 3.6, respectively, compared to those previously reported for Denmark and Greenland, 3.2 and 2.8, respectively. A_e_ values greater than 6 were found for eight loci (mh01KK-117, mh04KK-030, mh06KK-008, mh11KK-180, mh13KK-217, mh13KK-218, mh21KK-315, mh21KK-324) in the Somalis, and for six loci (mh02KK-134, mh11KK-180, mh13KK-213, mh13KK-218, mh21KK-315, mh21KK-320) in the Afghans. 

Significant *F*_ST_ distances were observed between the Somalis, Afghans, Danes, and Greenlanders (Supplementary Table S7), a finding that was not surprising given their distant geographical assignments. Greenland showed high *F*_ST_ value with all other populations, reflecting its intricate history of peopling and subsequent demographic relationships under a scenario of geographical isolation [34]. Overall, the highest genetic differentiation was observed between Greenland and Somalia (*F*_ST_ = 0.16389). By comparison, the Danes and Afghans exhibited the lowest *F*_ST_ value (*F*_ST_ = 0.05649). PCA and STRUCTURE analyses confirmed a greater genetic proximity between these two populations among the four populations (see below).

**Figure 1:**
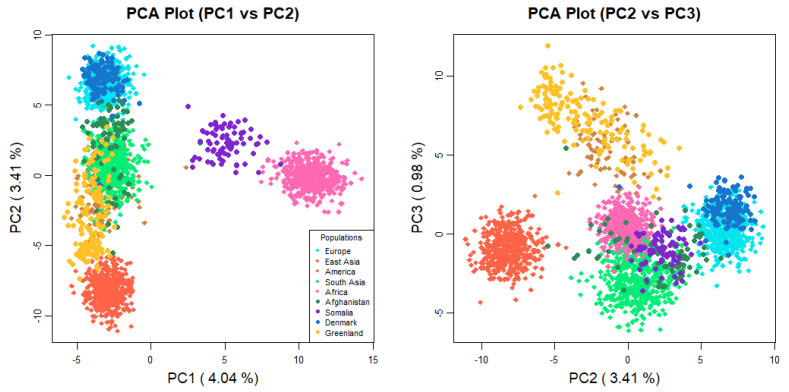
PCA plot with Afghan, Somali, Danish, and Greenlandic individuals. Additionally, 21 populations from 1000 Genomes Project database (African populations: GWD, ESN, MSL, YRI, LWK; European populations: GBR, CEU, FIN, IBS, TSI; South Asian populations: BEB, STU, ITU, PJL, GIH; East Asian populations: KHV, CDX, CHS, CHB, JPT; American population: PEL) were included in the analysis.

**Figure 2:**
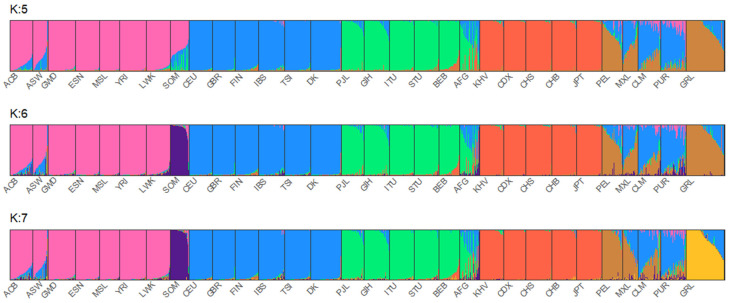
STRUCTURE results for K:5, K:6, and K:7. All 26 populations from 1000 Genomes Project were included in the analysis together with Afghanistan (AFG), Somalia (SOM), Denmark (DK), and Greenland (GRL).

The MH-74 plex has 30 loci that were retrieved from previous selections [35,36] due to their ancestry informativeness. In previous studies of four U. S. population groups [16], and in sub-Saharan Africans, Iraqis, and Germans [37], the MH-74 plex effectively distinguished between distinct major continental population groups. A PCA plot (Figure 1) was generated to assess both interpopulation structure and genetic proximity among the studied individuals. The 1000 Genomes Phase 3 individuals from 21 populations were included in the analysis (African populations: GWD, ESN, MSL, YRI, LWK; European populations: GBR, CEU, FIN, IBS, TSI; South Asian populations: BEB, STU, ITU, PJL, GIH; East Asian populations: KHV, CDX, CHS, CHB, JPT; American population: PEL). To simplify the interpretation of the plot, populations known to have experienced historical admixture were disregarded from this analysis, except for the Peruvian population, since it is the population with the highest Native American component [38]. In the PCA analysis, PC1 (4.04%) and PC2 (3.41%) elucidated 7.45% of the overall genetic variation. The Afghans were positioned closer to the Danes than the Greenlandic and Somali individuals. Moreover, compared to the other South Asians, the Afghans were plotted closer to the European populations. The STRUCTURE analysis (Figure 2) also points to a higher European co-ancestry in the Afghan population compared with the remaining South Asian populations included in the analysis. High East Asian co-ancestry was detected in some Afghan individuals, which is consistent with their distribution in the PCA plot. The Somalian individuals formed a cluster, which was close to other African individuals and far from Eurasians. The separation of the Somalis from the remaining sub-Saharan African individuals emerged when K:6 and K:7 were assumed in the STRUCTURE analysis. Previous studies using SNPs have also observed modern Somali individuals clustering together and slightly separated from the rest of the analyzed sub-Saharan African individuals [39]. It is important to acknowledge that both studies used similar sampling, without considering the possible different ethnicities of the Somali individuals. The visualization of the PC3 (0.98%) depicted separation of the Greenlandic individuals from the remaining populations. Additionally, the PCA plot illustrated the high intrapopulation diversity of the Afghans and Somalis, as both populations are distributed across a broad range in the plot. The interpretation is sustained by the genetic diversity levels captured in this study—0.68 in Afghans and 0.66 in Somalis—(Supplementary Table S4) and in previous studies based on autosomal DNA [40,41,42] and Y-chromosomal DNA [43] analyses of Afghans and Central Asian populations and autosomal DNA analyses of populations from the Horn of Africa [44].

### 3.4. Kinship Analyses

Simulations were performed in the Familias software [29] using allele frequencies from the Somali and Afghan populations (Supplementary Table S3). 

**Figure 3:**
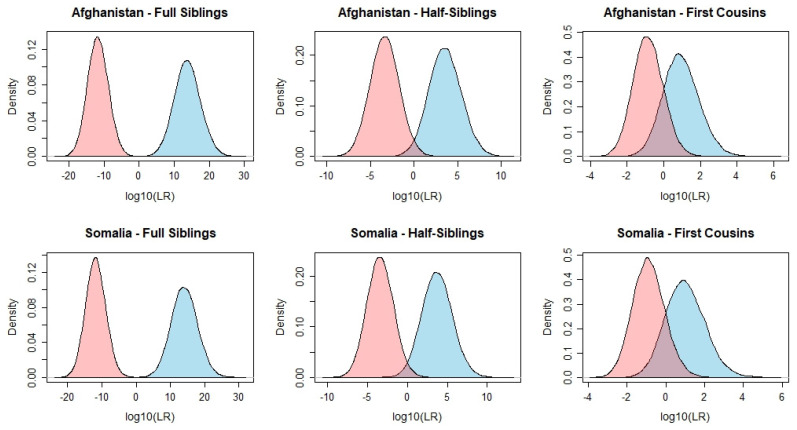
Log10(LR) distributions calculated for pairwise kinship scenarios. Blue curves: simulated full siblings, half-siblings, or first cousins. Pink curves: simulated unrelated pairs. For each kinship scenario, 30,000 simulations were computed.

The kinship simulations and corresponding LRs were calculated for different kinship scenarios. The main hypothesis was that the individuals were related as either full siblings, half-siblings, or first cousins, and the alternative hypothesis was that they were unrelated. Graphical representations of the obtained LR distributions (log10 scale) were generated for the above-mentioned kinship scenarios (Figure 3) as well as the calculation of the false discovery rate (FDR), i.e. false positive and false negative rates, using different LR thresholds (Supplementary Table S8). Given a specific threshold T, a false positive result occurred when two unrelated individuals were incorrectly identified as related, LR > T, while a false negative occurred when two related individuals were incorrectly identified as unrelated, LR < 1/T.

For simulated full sibling pairs, the LRs ranged from 0.06 to 1.22 × 10^29^ (median 4.95 × 10^13^) for the Afghans and from 0.02 to 4.60 × 10^30^ (median 1.13 × 10^14^) for the Somalis. When considering the unrelated individuals, the LRs ranged from 8.71 × 10^−24^ to 13.55 (median 1.52 × 10^−12^) for the Afghans and from 1.82 × 10^−23^ to 2.75 (median 1.40 × 10^−12^) for the Somalis. For both populations, a small overlap of the areas under the LR curves was observed (Figure 3), with LRs ranging from 0.06 to 13.55 in the Afghans and from 0.02 to 2.7 in the Somalis, including 0.01% and 0.02% of the cases, respectively. The FDR obtained for both kinship hypotheses and both populations was null for T ≥ 10^2^. 

For the kinship problem considering the hypotheses half-siblings (H1) and unrelated (H2), the LR distributions for the Somalis varied from 1.32 × 10^−4^ to 4.16 × 10^12^ (median 5.04 × 10^3^) and for the Afghans from 2.83 × 10^−4^ to 5.45 × 10^10^ (median 3.50 × 10^3^). Conversely, when considering individuals simulated as unrelated, the LR values varied from 1.29 × 10^−10^ to 8.69 × 10^3^ (median 4.40 × 10^−4^) and from 8.37 × 10^−11^ to 1.93 × 10^3^ (median 3.68 × 10^−4^) for Afghan and Somali individuals, respectively. An overlap between the areas under the LR distribution curves of the simulated half-sibling and unrelated pairs was observed for both populations (LRs ranging from 2.83 × 10^−4^ to 8.69 × 10^3^ in the Afghans and from 1.32 × 10^−4^ to 1.93 × 10^3^ in the Somalis), comprising 56.47% and 50.93% of the cases, respectively. For T = 10^4^, the FDR obtained showed to be null, reaching roughly 4% for a theoretical T = 1 (Supplementary Table S8). To mitigate these rates, the number of loci in the panel should be increased. Du et al. [45] concluded that for an LR threshold of a thousand, the set of MHs should comprise at least 300 loci, a number that varies depending on the average A_e_ and other statistical parameters of the set. 

Not surprisingly, of the 3 simulated kinship scenarios, the one involving the hypotheses first cousins (H1) and unrelated (H2) presented the lowest LR values and more overlap of the LR distributions between the two scenarios. When considering the individuals simulated as first cousins, the LR distributions ranged from 1.04 × 10^−3^ to 9.27 × 10^4^ (median 9.12) in the Somalis and from 3.37 × 10^−3^ to 1.17 × 10^6^ (median 8.20) in the Afghans. When considering individuals simulated as unrelated, the LR distributions ranged from 2.38 × 10^−4^ to 894.50 (median 0.12) in the Somalis and from 1.87 × 10^−4^ to 339.42 (median 0.14) in the Afghans. As expected, the FDR showed to be substantially higher for this kinship problem, scoring 15.13% and 14.27% of false positives and 16.20% and 15.97% of false negatives in the Afghans and Somalis, respectively, when considering the theoretical threshold T = 1. For T = 10^2^, these rates dropped to 0.05% of false positives and 0.03% of false negatives and to 0% for T ≥ 10^3^.

The substantial overlap between the areas under the LR distribution curves of first cousins and unrelated pairs emphasizes the necessity for complementing this panel with more MH loci or STRs. 

This is in line with the recent study conducted by Wu et al. [46] in a northern Han Chinese population, who concluded that even with approximately 400 MHs with an average H_e_ of ~0.7, distinguishing first cousins from unrelated individuals may still pose challenges. Other studies employing a similar number of loci demonstrated comparable FDRs that varied depending on the kits. A selection of 67 MHs with an average H_e_ = 0.735 and a CPE = 1–3.27 × 10^−25^ demonstrated lower FDRs (null for full siblings, 0.001 when T = 10^2^ and 0.0002 when T = 10^3^ for 2nd degree relationships (relationships not specified), and 0.008 when T = 10^2^ and 0.0008 when T = 10^3^ for first cousins/great uncle-nephew relationships) in southwestern Han Chinese individuals [47]. In the same study, Xue et al. also tested a panel with only 53 MHs but with a CPE = 1–2.61 × 10^−27^. The 53 MH assay revealed even lower FDRs than the 67 MHs set, suggesting that refining the locus selection to fewer, yet more informative, loci could enhance the kinship analysis. Despite not providing information on FDRs, another panel containing 60 MHs with an average H_e_ = 0.727 and a CPE = 1–4.78 × 10^−18^ showed lower average LRs (1.48 × 10^9^ for full siblings and 8.91 × 10^2^ for grandfather–grandson/half-siblings/uncle–nephew relationships) than the ones reported in our study [48].

Previous studies using panels of STRs or SNPs, or a combination of both, have also tested the efficiency of these genetic markers for kinship analyses [18,49,50]. In all the studies where both types of genetic markers were evaluated, despite presenting fewer loci, STRs exhibited higher efficiency than SNPs. The main reason is the multi-allelic nature of the STRs, leading to sets with higher A_e_ values compared with SNP panels, which typically present low A_e_ values. The STR sequences obtained by MPS technologies present an even higher discrimination power due to the higher number of alleles compared to the PCR-CE analyses [51,52]. Nonetheless, the high mutation rate of STRs may lead to ambiguous conclusions that may require supplementary investigations with other markers [49,52]. SNPs, which are intrinsically more stable and characterized by lower mutation rates, are often seen as a good alternative in cases where a possible mutation in an STR locus has led to equally likely kinship scenarios [49]. MHs are a multi-SNP genetic locus that provide greater stability than STRs and allow the development of panels with high overall A_e_ value. In a validation study made in-house, it was observed that STR and SNP panels presented lower LRs and higher FDRs than the MH-74 plex panel [18]; consequently, the MH-74 plex has been implemented as a supplementary investigation in our laboratory. 

## 4. Conclusions

In this study, we attest to the versatility of MHs, which represent the most recent generation of genetic loci suitable for forensic applications. Sequencing 74 MHs in individuals originating from Somalia and Afghanistan disclosed a substantial number of polymorphisms that were not part of the original MH allele-defining SNPs. The addition of these polymorphisms enhanced the MH informativeness and elevated the usability of the MH-74 plex panel. The intrapopulation diversity parameters revealed higher average A_e_, CPD, and CPE values in Somalia and Afghanistan than in the previously studied populations from Denmark, Greenland, the U.S., and China, attesting to the applicability of the MH-74 for identification purposes and mixture deconvolution in these populations. The MH-74 plex panel also demonstrated efficacy for ancestry analysis, achieving high resolution in the clustering of the analyzed populations. Nevertheless, an MH panel exclusively comprising ancestry-informative MHs would likely enhance the clustering accuracy further, opening the prospect for the future development of an ancestry-focused MH panel. In addition to being useful for population genetic studies, such a panel could also aid in forensic contexts, e.g., inferring the biogeographic ancestry of trace samples. 

Simulations of four different degrees of relatedness showed the distinct performance of this set of 74 MHs for kinship assessments. For full siblings vs. unrelated kinship analyses, the MH-74 plex demonstrated good performance with high LRs and extremely low false positive and negative rates, whereas it was unable to identify half-siblings or cousins from unrelated pairs of individuals. Thus, a more extensive number of loci and better loci are required to reach statistically significant LR results for kinship analyses, in which hypotheses involving more distant relationships than full siblings are compared with unrelated individuals. This study highlighted the potential of MHs for human identification, ancestry inference, and kinship analysis. However, further research on MHs is needed, namely by developing distinct panels that are specifically tailored for different forensic purposes. 

## Data Availability

The data presented in this study are available in article and supplementary material.

## Supplementary Materials

The following supporting information can be downloaded at: https://www.mdpi.com/article/10.3390/genes16050532/s1, Table S1: List of microhaplotypes where the default noise filter and/or slide function were changed; Table S2: Variants identified in Somalis and Afghans in the MH-74 plex that were not part of the original MH configuration; Table S3: Allele frequencies in Afghans and Somalis for 72 microhaplotypes; Table S4: Forensic and population parameters (alleles called per locus, allele count per locus, polymorphism information content, power of discrimination, probability of exclusion, observed and expected heterozygosity, and effective number of alleles) for Afghans and Somalis; Table S5: Hardy-Weinberg equilibrium after Bonferroni correction in Afghans and Somalis; Table S6: Comparison between CPE, CPD, and average PIC and H_e_ across Afghan, Somali, Danish, Greenlandic, Chinese, and U.S populations; Table S7: Pairwise FST values calculated for all population pairs based on 72 MHs; Table S8: Percentages of simulations, where related and unrelated individuals were correctly assigned *, incorrectly assigned †, or the results were inconclusive ‡ assuming different LR thresholds (T = 1; T = 10; T = 10^2^; T = 10^3^; T = 10^4^).
